# Geographical access to point-of-care testing for hypertensive disorders of pregnancy as an integral part of maternal healthcare in Ghana

**DOI:** 10.1186/s12884-020-03441-6

**Published:** 2020-11-25

**Authors:** Desmond Kuupiel, Kwame Manu Adu, Vitalis Bawontuo, Philip T. N. Tabong, Duncan A. Adogboba, Tivani P. Mashamba-Thompson

**Affiliations:** 1 Department of Public Health Medicine, School of Nursing and Public Health, University of KwaZulu-Natal, Durban, South Africa; 2 Research for Sustainable Development Consult, Sunyani, Ghana; 3 Department of Geography, University of Ghana, Legon, Ghana; 4 Faculty of Health and Allied Sciences, Catholic University College of Ghana, Fiapre, Sunyani Ghana; 5 School of Public Health, University of Ghana, Legon, Ghana; 6 Regional Health Directorate, Ghana Health Service, Upper East Region, Bolgatanga, Ghana

**Keywords:** Accessibility, Point-of-care testing, Screening, Hypertensive disorders of pregnancy, Antenatal care, Primary healthcare clinics, Upper East Region, Ghana

## Abstract

**Background:**

Hypertensive disorders of pregnancy (HDP) are associated with high maternal mortality in Ghana and globally. Evidence shows that there is poor availability of pregnancy-related point-of-care (POC) tests in Ghana’s primary healthcare (PHC) clinics (health centre or community-based health planning services facilities). Therefore, we employed geographic information systems to estimate the geographical distribution of and physical accessibility to HDP POC testing services in the Upper East Region (UER), Ghana.

**Methods:**

We collected data on 100 out of 365 PHC clinics, public hospitals providing HDP testing, PHC clinic type, ownership, and availability of urine dipsticks and blood pressure (BP) devices. We also obtained the geo-located data of the PHC clinics and hospitals using the global positioning system. We employed ArcGIS 10.4 to measure the distance and travel time from the location of each PHC clinic without HDP POC testing services as well as from all locations of each district to the nearest hospital/clinic where the service is available. The travel time was estimated using an assumed motorised tricycle speed of 20 km/hour. We further calculated the spatial distribution of the hospitals/clinics providing HDP POC testing services using the spatial autocorrelation tool in ArcMap, and Stata version 14 for descriptive statistical analysis.

**Results:**

Of the 100 participating PHC clinics, POC testing for HDP was available in 19% (14% health centres and 5% community-based health planning services compounds) in addition to the 10 hospitals use as referral points for the service. The findings indicated that the spatial pattern of the distribution of the health facilities providing HDP POC testing was random (z-score = -0.61; p = 0.54). About 17% of the PHC clinics without HDP POC testing service were located > 10 km to the nearest facility offering the service. The mean distance and travel time from PHC clinics without HDP POC testing to a health facility providing the service were 11.4 ± 9.9 km and 31.1 ± 29.2 min respectively. The results suggest that if every 19% of the 365 PHC clinics are offering HDP POC testing in addition to these 10 hospitals identified, then the estimated coverage (health facility-to-women in fertility age ratio) in the UER is 1: 3,869.

**Conclusions:**

There is poor physical accessibility to HDP POC testing services from PHC clinics without HDP POC testing in the UER. Mothers who obtain maternal healthcare in about 17% of the PHC clinics travel long distances (> 10 km) to access the service when needed. Hence, there is a need to improve the availability of HDP POC diagnostic tests in Ghana’s rural clinics.

## Background

Hypertensive disorders of pregnancy (HDP) are a group of disorders that includes chronic hypertension ( arising before 20 weeks’ gestation or persisting longer than 12 weeks after delivery), gestational hypertension (arising after 20 weeks’ gestation), preeclampsia or preeclampsia superimposed on chronic hypertension [1–4]. Generally, hypertensive disorders contribute significantly to maternal mortality, prolonged disability, and perinatal death [5, 6]. In 2014, a World Health Organization (WHO) systematic review evinced that hypertensive disorders accounted for about 14% of the 60 799 total maternal deaths globally from 2003 to 2009 [7]. It is estimated that preeclampsia alone is accountable for more than 500 000 fetal and neonatal deaths and over 70 000 maternal deaths annually in the world [2]. Approximately 99% of these deaths occur in low-and-middle-income countries (LMIC) with Sub-Saharan Africa accounting for close 64% [6–9].

Preeclampsia is diagnosed as elevated blood pressure in pregnancy accompanied by either new-onset proteinuria or a severe feature such as acute kidney injury, liver involvement, neurological complications, haematological complications, and uteroplacental dysfunction [2, 3, 10, 11]. Of recent, proteinuria is no longer essential for the diagnosis of preeclampsia according to the International Society for the Study of Hypertension in Pregnancy (ISSHP) [2, 3]. To facilitate early detection and diagnosis of preeclampsia, ISSHP recommends blood pressure (BP) monitoring of women at each antenatal care (ANC) visit, urine test for protein if the blood pressure is high, and symptoms as headache, visual disturbance, and epigastric pain using visual dipstick testing according to the manufacturer’s specification. ISSHP also recommends that every ANC clinic/unit should have as a minimum a dedicated sphygmomanometer and urine dipsticks for detecting proteinuria [2] It further recommends training of healthcare workers on how to measure BP appropriately using the correct technique [2].

In Ghana, the current maternal mortality ratio (MMR) is 319 per 100 000 live births according to the WHO 2018 world health statistics report [9] which is still much higher than the 70 per 100,000 live births targeted by sustainable development goal 3.1. Recent studies in Ghana have revealed hypertensive disorders such as chronic hypertension and pre-eclampsia/eclampsia as the leading cause of maternal deaths ahead of haemorrhage in recent times accounting for approximately 26.7% [12, 13]. To this end, screening of women for hypertensive disorders during ANC at first and subsequent visits is a requirement in all healthcare facilities particularly, for expectant mothers with a previous history of pre/eclampsia or high BP [14, 15]. Healthcare services or provisions in Ghana are administered at five levels (National, Regional, District, Sub-district, Community level). In Ghana, primary healthcare facilities (health centres and community-based health planning and services (CHPS) compounds) located at the sub-district and community levels most often lack laboratories although they provide maternal health services such as ANC, delivery, and post-natal care. These healthcare facilities are mostly led by a Midwife or Physician Assistant (Clinical) or a Nurse Assistant (Preventive or Clinical). In Ghana, the diagnosis of hypertensive disorders such as preeclampsia at the health centre and CHPS compound levels is mostly made primarily based on BP, symptoms, and proteinuria and referred to the nearest district hospital for further laboratory investigations and care when needed.

Despite this, previous studies in the Upper East Region (UER) of Ghana demonstrated low availability, weak supply chain management, and stock-outs of pregnancy-related point-of-care (POC) tests including urine dipsticks for proteinuria and BP monitoring devices [16–18]. Previous research that evaluated geographical access to POC testing services focused on Tuberculosis [19], Glucose-6-Phosphate Dioxygenase Deficiency [20], and Blood Group and Rhesus Type [21]. However, to date, no study has measured the physical accessibility (distance and travel) to healthcare facilities where a test for HDP such as preeclampsia is available in the UER, Ghana, worldwide. Evidence-based information on the physical accessibility of the service may influence policymakers and implementers to facilitate the implementation of POC testing services for HDP in poor access areas. We, therefore, sought to answer the question “what is the geographical distribution of and physical accessibility to HDP POC testing in the UER of Ghana?” Firstly, we described the availability and the geographical distribution of the health facilities providing the services in the UER, and secondly, we measured the physical accessibility to HDP POC testing service as part of ANC in the UER, Ghana.

## Methods

### Study design/area

We employed geographic information systems to estimate the geographical distribution of and physical accessibility to HDP POC testing services as an integral part of ANC in the UER of Ghana based on the findings of a previous cross-sectional survey [18]. The cross-sectional study before the current study found poor availability of pregnancy-related POC diagnostic tests in the UER [18]. A detailed description of the study setting has been described and published elsewhere [17–19].

### Data collection

The study was conducted from February to March 2018 in all 13 districts using a random sample of 100 out of a total 365 public PHC clinic providing maternal healthcare services from all the districts. A multistage sampling strategy involving stratified, probability proportionate to size, and simple random sampling techniques. A detailed description of this study’s sampling strategy is published elsewhere [18]. We obtained the geographic information (geo-located data) of the PHC clinics and their referral health facilities from the Upper East Regional Health Directorate, and the use of a global positioning system. We then applied the world geodetic system Zone 30 north coordinate system to all spatial data to allow for the results of spatial processes in a chosen unit of meters. Topographic data included roads, rivers, and the digital elevation model. The collectors interviewed the midwives/nurses in-charge of the antenatal clinics or the heads of the clinics for information about the clinic type, ownership, availability of urine dipsticks, BP monitoring devices, and name of the nearest hospital (referral facility) and geographical location (Town/village) using a questionnaire (published elsewhere) [18]. We also cross-checked the information about the referral health facilities to be sure of the availability of urine dipsticks for proteinuria testing and BP monitoring device. To ensure data quality, the principal investigator closely supervised the data collection and data entry activities for consistency and completeness of information throughout the study period.

### Variables and operational definitions

#### Availability

The availability of urine dipsticks for proteinuria testing and BP monitoring devices, or laboratory services.

#### Distance

Proximity to the nearest health facility where a POC testing service for HDP is available.

#### Travel time

Estimated time likely to be spent by an expectant mother traveling from a PHC clinic or her settlement location to the entrance of a health facility providing HDP diagnostic services.

### Data analysis and mapping

We used PHC clinics that lacked urine dipsticks and BP monitoring devices from all 13 districts, as inputs to measure proximity to the nearest health facilities providing HDP testing services. We computed the Euclidean distance from the PHC clinic to the nearest HDP diagnostic services using the near function of analysis tools in ArcGIS 10.4 software. Data on the health facilities, area, and the geographic coordinates of the PHC clinics and their referral health facilities were linked to ArcGIS 10.5 software and a base map. Travel time was estimated using a motorised tricycle transport system (“motor king”) since it is the most used public transport in the UER. The model and procedure used to estimate the travel time for this study have been published elsewhere [19]. We employed ArcMap 10.5. to calculate the spatial autocorrelation or Moran’s Index (MI) of the health facilities providing HDP diagnostic services and, the z-score and p-value reported. MI value of 0, or very close to 0, was considered as random distribution, MI value less than zero was interpreted as dispersed distribution, and MI value greater than zero was considered as a clustered distribution. Data on the distance and travel time to the nearest health facility providing HDP diagnostic services from the clinics were exported to Stata version 14.0 and the mean distance and travel time calculated for each district. We considered PHC clinics located > 10 km from the nearest health facility providing HDP POC testing services as areas with poor physical accessibility. Research has shown that physical access to healthcare beyond 10 km is significantly associated with higher risks of poor health outcomes [22].

## Results

### Availability and geographical distribution of health facilities providing HDP POC testing in the UER

Of the 100 participated PHC clinics, POC testing for HDP was available in 19% (14% health centres and 5% CHPS compounds) in addition to the 10 hospitals (all 7 District hospital, the Regional hospital, and 2 private hospitals) use as referral points for the service. Of these 29 health facilities providing HDP POC testing services, 6 (21%) were in the Garu-Tempane District, 4 (14%) in the Bongo district, and 3 in the Bolgatanga Municipality. Whilst 2 (7%) health facilities each were geo-located in the Bawku Municipal, Kasena Nankana Municipal, Bawku West, Builsa North, Builsa South, Kasena Nankana West, and Pusiga districts. Only 1 (3%) health facility each providing HDP POC testing was geo-located in the Nabdam and Talensi Districts. Twenty-two (76%) out of the 29 health facilities providing HDP POC testing services were owned and managed by the Ghana Health Service. Five (17%) were owned and managed by the Christian Health Association of Ghana, and 2 (7%) private facilities. Based on this result, HDP POC testing service coverage (health facility-to-women in fertility age ratio) in the UER was estimated to be approximately 1:3,869 if every 19% of the total 365 PHC clinics were offering HDP POC testing in addition to these 10 hospitals identified as referral facilities for the participating PHC clinics, and the estimated 305,683 women in fertility age in the region.

**Fig. 1:**
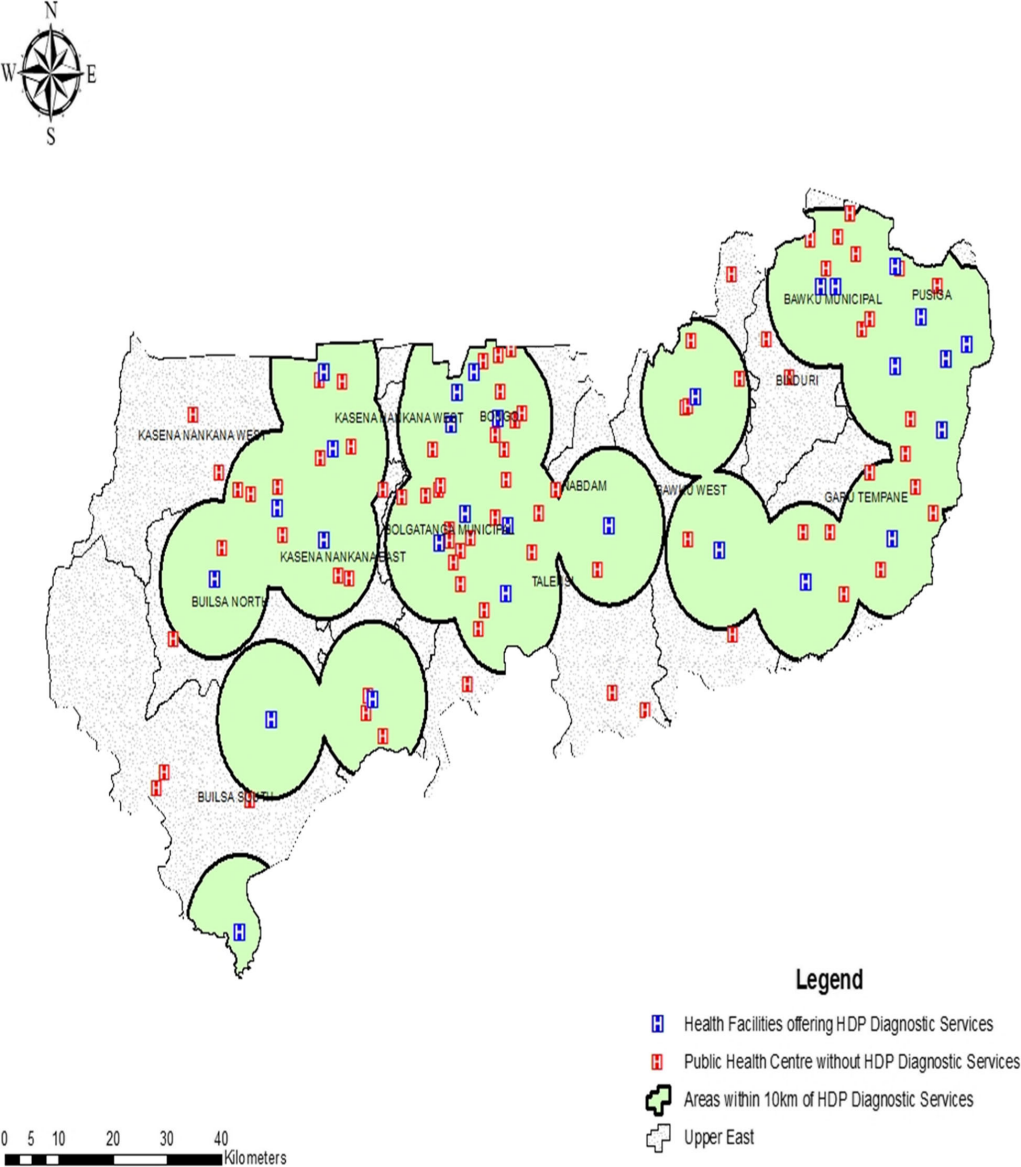
Geographical distribution of health facilities providing HDP POC testing and clinics without the service and their geo-location within 10 km range from the nearest health facility providing the service in the region

Of the 81 PHC clinics where HDP screening services were not available, 53 (65%) were CHPS compounds, whilst 28 (35%) were health centres. Of these 81 clinics, 10 (12%) facilities each were geo-located were in the Bongo and Garu-Tempane districts; 9 (11%) clinics each were in the Bawku West, Kasena Nankana West, and Bolgatanga municipal. Whilst 7 (9%) clinics each were geo-located in the Kasena Nankana Municipality and Talensi district; 5 (6%) located in the Bawku Municipality; and 4 (5%) in the Binduri district. Three (4%) each were also found in the Builsa South and Pusiga districts, while 2 (3%) of the 81 clinics were geo-located in the Nabdam districts. Out of the 81 clinics, 77 (95%) were owned by and managed by Ghana Health Service, and 4 (5%) were Christian Health Association of Ghana facilities. Figure 1 presents a map describing the geographical distribution of the 29 facilities providing HDP POC testing and the 81 clinics (without HDP POC testing services) and their geo-location within 10 km range from the nearest health facility providing the service in the UER.

### Spatial distribution of the health facilities providing HDP diagnostic services

**Fig. 2:**
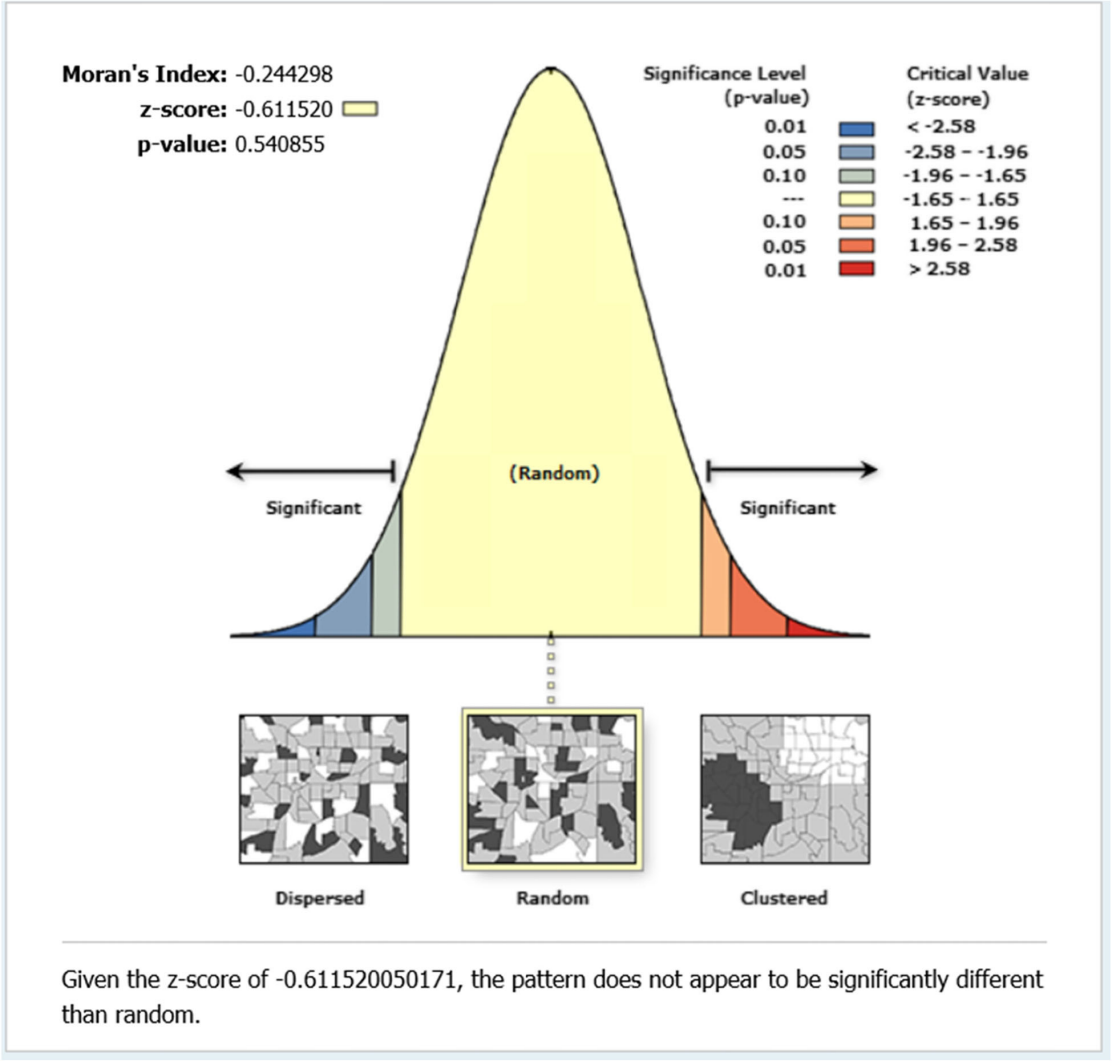
Output of the spatial autocorrelation of health facilities providing HDP POC testing services in the region

We conducted a spatial autocorrelation to measure the closeness of the health facilities where HDP POC testing services were available in the region. The results showed an MI value closed to zero (-0.24). This MI result indicates the health facilities providing HDP POC testing services in the UER are randomly distributed spatially (z-score = -0.61; *p* = 0.54). Figure 2 presents the output of the spatial autocorrelation analysis.

### Physical accessibility to HDP POC testing service in the UER

The results showed that about 83% (67/81) of the clinics without HDP POC testing services were within 10 km proximity to the nearest health facility providing the services. The mean distance and travel time from the clinics without HDP POC testing services to the nearest health facility providing the services were 11.4 ± 9.9 km and 31.1 ± 29.2 min respectively. Whilst, the mean distance and travel time to a health facility providing HDP POC testing services used by the clinics without the services as referral centres were 17.3 ± 7.4 km and 52 ± 49 min respectively. Clinics without HDP POC testing in the Builsa South district recorded the longest mean distance (29.8 ± 1.5 km) and travel time (65.8 ± 57.9 min), while the shortest mean distance was recorded in the Bongo district (4.3 ± 2.0 km) and travel time (13.6 ± 5.9 min).

## Discussion

**Fig. 3:**
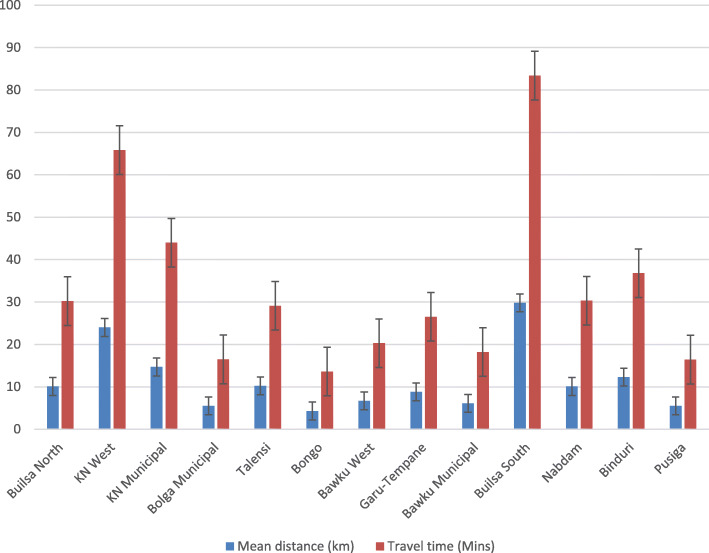
A clustered column chart comparing distance and travel time values across the districts in the UER.

This study assessed the geographical distribution of and physical accessibility to HDP POC testing services as an integral part of ANC in the UER, Ghana. Our study findings showed the majority (81%) of the participating PHC clinics were not providing HDP POC testing services due to the unavailability of urine dipsticks and BP monitoring devices. The MI value (-0.24) of the health facilities providing HDP POC testing services suggested the health facilities were distributed at random spatially. Also, the results showed that more than half (approximately 83%) of the participating PHC clinics without urine dipsticks and BP monitoring devices were located within 10 km proximity to the closest health facility providing HDP POC testing services in the region. However, physical accessibility (distance and travel time) from PHC clinics without HDP POC testing services was shown to be poor and varied across the 13 districts (Fig. 3).

Our study found that HDP POC testing services were not available in most of the participating PHC clinics in the UER. This revelation supports the findings of a previous study in Ghana which aimed to assess the accessibility of pregnancy-related POC diagnostic tests [18]. Similarly, a study aimed to audit the supply chain management of POC diagnostics in PHC clinics in Ghana also reported the stock-out of POC diagnostics [17]. The spatial pattern of the distribution of the health facilities providing HDP POC testing was revealed to be random. This implies no pattern at all with a tendency toward cluster or disperse. Hence, the need to make HDP testing services available in PHC clinics across the region. This supports the study finding similar studies that assessed the distribution of health facilities in the United Arab Emirates, and Nigeria [23, 24]. Our study also demonstrated poor physical access to facilities providing HDP POC testing services although the geographical distribution of the PHC clinics without HDP POC testing services suggested most of them were located within 10 km of a facility providing the services. Similarly, Tanser et al. found poor physical accessibility (travel time of 170 min) to healthcare in South Africa [25]. We also found a difference (almost 6 km) between the average distance as well as the travel time (20.9 min using the motor king transport system) to the closest facility providing HDP POC testing services and the choice of the health facilities used by PHC clinic health providers as referral centres. It may be that healthcare providers at those clinics prefer to refer the mothers to a health facility where they can additionally access most other diagnostic tests probably not available in their facilities yet essential for ANC.

Although availability, distance, and travel time are not the only determinants of access to healthcare, the implications of our study findings could influence utilisation and timeliness of HDP diagnosis in the region [19, 21, 26–28]. Poor accessibility to health services such as in this study, most often result in longer distance and increase travel time to the POC. It may also result in a higher cost of transportation and other unforeseen expenditures in accessing health facilities providing HDP POC testing services in the UER [19]. Also, waiting time at facilities rendering HDP POC testing services may increase if pregnant mothers in an area crowd in one facility for testing [29]. Consequently, this situation may lead to low patronage, poor utilization, late detection of HDP, and late initiation of treatment or referral among others [27] since people most often tend to limit the use of healthcare services closer to them [30].

It is worthwhile to improve the availability of HDP POC tests in all PHC clinics in the region but first, targeting PHC clinics located greater than 10 km from the nearest facility offering HDP POC testing such as those located in the Builsa South, Kassena Nankana West, Kassena Nankana Municipal, and Binduri districts where we found the participating clinics to have poorer physical accessibility. It is our opinion that the WHO-approved urine dipsticks for proteinuria should be made available in all/most PHC clinics [31]. BP monitoring devices should also be made available in all clinics at the PHC to enable POC testing and early detection of hypertensive disorders among pregnant during ANC. Furgerson et al. study demonstrated having POC diagnostic tests closer to people streamlines critical care paths [29]. We further recommend the strengthening of supply chain management of these diagnostic devices to prevent stock-out and ensure the sustainability of HDP POC testing services in rural PHC clinics [16, 32]. Subsequently, this could potentially lead to improved maternal and perinatal health outcomes, and reduce maternal mortality to less than 70 per 100,000 live births as stipulated by sustainable development goal 3.1 [33]. Moreover, we encourage the Ghanaian Ministry of Health to establish an essential diagnostic list (EDL) as recommended by the WHO [31]. Establishing and implementing the EDL potentially could improve access to health services and accelerate Ghana’s achievement of most of the health-related sustainable development goals by 2030 including universal health coverage.

Our study findings could support policy or decision-makers to appreciate the variations in physical accessibility to HDP POC testing services across the region and its potential relationship with maternal mortality caused by HDP in Ghana, which could help improve the provision of HDP POC testing services in PHC clinics. Generally, measuring physical accessibility using GIS could help assess the distribution of and access to healthcare services [34, 35]. A major strength of this study is that it used geographical information systems (GIS) to provide evidence to the healthcare system in the UER using the HDP POC testing services as a proxy pointer to assess the physical accessibility which covered the entire region to measure distance and travel time from health facilities. The application of GIS for this study also enabled us to identify HDP POC testing service disparities and poor geographic access areas in the UER. Despite these strengths, a major limitation of this study was our inability to include non-spatial variables such as socioeconomic factors and other maternal factors in the model since we did collect that data. Again, data was collected from only 100 out of 365 PHC clinics, so it is possible some PHC clinics, as well as other privately owned clinics and medical laboratories existing in the region not included in this study, maybe providing HDP testing services. Hence, the distances and travel times may be lower than estimated if all clinics and private health facilities offering HDP POC testing were included in this study’s analysis. We, therefore, suggest that this study’s findings should be interpreted with caution. Nonetheless, we estimated the coverage for HDP testing service in the region to be 1: 3,869 assuming every 19% of the 365 PHC clinics in addition to the 10 hospitals identified as referral points were providing HDP POC testing. We estimated travel time using only one mode of transportation which may not apply in all cases since other choices such as bicycle, motorbike, walking may be used by expectant mother to HDP testing service provision facilitates. This study was conducted using only one region in Ghana hence, the generalizability of the findings may be limited. Regardless of these limitations, this study provided valuable information from the available data to help improve HDP POC testing services in the region.

## Conclusions

The results of this survey show there is poor physical accessibility to HDP POC testing services from PHC clinics without HDP POC testing in the UER of Ghana. About 17% of the PHC clinics without HDP POC testing service were located greater than 10 km from the nearest facility offering the service. This implies that mothers who obtain maternal healthcare in those PHC clinics would have to travel long distances to access the service when needed. Hence, there is a need to improve the availability of HDP POC diagnostic tests and BP monitoring devices in rural clinics to facilitate prompt detection of women with hypertensive disorders and link them promptly to care. This potentially will contribute to a reduction of maternal deaths associated with HDP in the UER and Ghana as a country.

## Data Availability

Data from this study cannot be shared publicly because it contains sensitive, identifying health facilities information. All interested researchers/readers/persons who meet the criteria for access to confidential data can access the data set from the first author via this email address: desmondkuupiel98@hotmail.com or KuupielD@ukzn.ac.za. Data access may also be requested from the University of KwaZulu-Natal Biomedical Research Ethics Committee (BREC) from the following contacts: The Chairperson Biomedical Research Ethics Administration Research Office, Westville Campus, Govan Mbeki Building University of KwaZulu-Natal P/Bag X54001, Durban, 4000 KwaZulu-Natal, South Africa Tel.: +27 31 260 4769 Fax: +27 31 260 4609 Email: BREC@ukzn.ac.za.
